# *C9orf72* proline-arginine dipeptide repeats disrupt the proteasome and perturb proteolytic activities

**DOI:** 10.1093/jnen/nlad078

**Published:** 2023-10-03

**Authors:** Yifan Zhang, Sophia C K Nelson, Ashley P Viera Ortiz, Edward B Lee, Robert Fairman

**Affiliations:** Department of Biology, Haverford College, Haverford, Pennsylvania, USA; Department of Biology, Haverford College, Haverford, Pennsylvania, USA; Translational Neuropathology Research Laboratory, Department of Pathology and Laboratory Medicine, Perelman School of Medicine, University of Pennsylvania, 613A Stellar Chance Laboratories, Philadelphia, Pennsylvania, USA; Translational Neuropathology Research Laboratory, Department of Pathology and Laboratory Medicine, Perelman School of Medicine, University of Pennsylvania, 613A Stellar Chance Laboratories, Philadelphia, Pennsylvania, USA; Department of Pathology and Laboratory Medicine, Perelman School of Medicine, University of Pennsylvania, Philadelphia, Pennsylvania, USA; Department of Biology, Haverford College, Haverford, Pennsylvania, USA

**Keywords:** ALS, *C9orf72*, *Drosophila melanogaster*, Proteasome

## Abstract

The hexanucleotide G_4_C_2_ repeat expansion in *C9orf72* is the most frequent genetic cause of familial amyotrophic lateral sclerosis (ALS). Aberrant translation of this hexanucleotide sequence leads to production of 5 dipeptide repeats (DPRs). One of these DPRs is proline-arginine (polyPR), which is found in *C9orf72-*expanded ALS (C9ALS) patient brain tissue and is neurotoxic across multiple model systems. PolyPR was previously reported to bind and impair proteasomes in vitro. Nevertheless, the clinical relevance of the polyPR-proteasome interaction and its functional consequences in vivo are yet to be established. Here, we aim to confirm and functionally characterize polyPR-induced impairment of proteolysis in C9ALS patient tissue and an in vivo model system. Confocal microscopy and immunofluorescence studies on both human and *Drosophila melanogaster* brain tissues revealed sequestration of proteasomes by polyPR into inclusion-like bodies. Co-immunoprecipitation in *D. melanogaster* showed that polyPR strongly binds to the proteasome. In vivo, functional evidence for proteasome impairment is further shown by the accumulation of ubiquitinated proteins along with lysosomal accumulation and hyper-acidification, which can be rescued by a small-molecule proteasomal enhancer. Together, we provide the first clinical report of polyPR-proteasome interactions and offer in vivo evidence proposing polyPR-induced proteolytic dysfunction as a pathogenic mechanism in C9ALS.

## INTRODUCTION

Amyotrophic lateral sclerosis (ALS) is a severe and progressive neurodegenerative disorder that primarily affects motor neurons in both the brain and spinal cord, causing approximately 6000 new cases each year (1). As these motor neurons degenerate, voluntary muscle action is lost, leading to difficulty moving, speaking, swallowing, and breathing. Currently, ALS is incurable, and patients usually die 3–5 years post-diagnosis. Thus, it is crucial to investigate the molecular mechanisms underlying disease pathology in hopes of designing effective treatments, ameliorating disease burden, and increasing our overall understanding of ALS etiology.

Research has identified a hexanucleotide G_4_C_2_ repeat expansion in the *C9orf72* gene as the most frequent genetic cause of familial ALS; this familial form is referred to as C9ALS (2). Patients who manifest the disease have hundreds to thousands of these repeats while unaffected individuals have less than 23 (2, 3). The exact link between these repeats and disease pathology is unclear; however, several mechanisms have been proposed, including haploinsufficiency, production of aberrant RNA foci, and the generation of potentially toxic aggregative dipeptide repeat (DPR) proteins (3–5). The function of *C9orf72* is still under investigation but studies have proposed roles in nucleocytoplasmic trafficking, endosomal trafficking, autophagy regulation, and Rab-mediated cellular trafficking and protein degradation (6).

Despite being located in an intron of the gene, the nucleotide sequence encoding the DPRs is expressed through a process known as repeat-associated non-ATG (RAN) translation, which can begin at any point without the presence of a start codon (4, 7). This translation happens in both sense and antisense directions and results in the formation of 5 different DPRs containing the following dipeptide sequences: glycine-alanine (GA), glycine-proline (GP), glycine-arginine (GR), proline-alanine (PA), and proline-arginine (PR) (4). Of the DPRs, polyPR sequences have shown high toxicity across multiple model systems (4, 8, 9). Work done in vitro using cell cultures found that polyPR was potently neurotoxic to primary motor, cortical, and hippocampal neurons (9). Transgenic mouse models expressing polyPR in neurons show similar results, with polyPR-positive mice displaying motor imbalances, decreased brain weight, inflammation in the cerebellum and spinal cord, and significant loss of lower motor neurons, hallmark manifestations of ALS (8). In *Drosophila* models, expression of polyPR reduces survival and locomotive ability (4). Both the precise localization pattern of polyPR and its mechanisms of toxicity are still under investigation, but it has been shown in part to aggregate and localize in the nucleus and nucleolus (8, 9).

Interestingly, ubiquitinated inclusions have been observed in motor neurons of C9ALS patients, suggesting that dysfunction of the proteostasis network may play a role in disease etiology (10). Dysfunction of this network has been linked to a wide variety of diseases and conditions such as cancer, autoimmune disorders, and neurodegenerative disease (10–12). Given the crucial role in homeostasis and health of the ubiquitin-proteasome system (UPS), modulation of the proteostasis network in cells may hold promise as a therapeutic avenue in diseases that involve protein misfolding and aggregation such as ALS. Supporting this theory, in vitro studies of polyPR have highlighted proteasome inhibition as a possible mechanism of toxicity (13–16). A study investigating the effects of polyPR expression in murine motor neuron cell culture found that polyPR was localized in nuclei while also directly associated with proteasomes. This resulted in reduced proteasome activity, inhibition of substrate degradation, and motor neuron death (15). In another key study, researchers using a synthetic PR-repeating peptide demonstrated a requirement for at least 2 positively charged residues (such as arginine) and proximal hydrophobic residues thereby providing a likely molecular explanation for why polyPR and polyGR have differential effects on proteasome function (13, 15). While the molecular nature of polyPR disruption of the proteasome is unknown, biochemical studies have shown that PR-rich peptides can bind to and destabilize the α-ring of the proteasome, interfering with the formation of both 20S and 26S proteasomes, and impeding substrate degradation (13, 14, 17, 18). Genetic disruption of proteasome genes in cell culture further supports an interaction, as such disruptions can modulate C9ALS-like degeneration and polyPR toxicity (16, 19). In this work, we tested for evidence of a polyPR-proteasome interaction in both human disease tissue and in a transgenic *Drosophila* model system expressing a polyPR50 DPR (9). Further, we explore the impact of such an interaction on the function of the proteasome in *Drosophila*.

## MATERIALS AND METHODS

### Human tissue immunofluorescence

Dual-immunofluorescence was performed on 6-µm-thick formalin-fixed paraffin-embedded postmortem cerebellum tissue sections from 7 cases with the *C9orf72*-expansion mutation. Samples were acquired from and genotyped at the Center for Neurodegenerative Disease Research brain bank at the University of Pennsylvania. Tissue sections were deparaffinized and rehydrated followed by antigen retrieval by microwaving in a citrate-based antigen unmasking solution (Vector Laboratories, Newark, CA, Cat No. H-3300). Sections were blocked in 2% fetal bovine serum (FBS) in 0.1 M Tris buffer and incubated with primary antibodies against poly-PR [anti-*C9orf72*/C9RANT (poly-PR), rabbit polyclonal, Sigma Aldrich, dilution 1:200] and proteasome subunit alpha type-1 (PSMA1) [anti-PSMA1, mouse monoclonal (OTI9H1), ThermoFisher, Waltham, MA, dilution 1:200], overnight at 4°C in a humidified chamber. Sections were washed in 0.1 M Tris buffer, blocked in 2% FBS in 0.1 M Tris buffer, and incubated with secondary antibodies (Alexa Fluor 488-conjugated goat anti-rabbit IgG; Alexa Fluor 647-conjugated goat anti-mouse IgG) for 2 hours at room temperature in a humidified chamber. Sections were washed in 0.1 M Tris buffer, stained using 4′-6-diamino-2-phenylindole (DAPI) to visualize nuclei (300 nM concentration, ThermoFisher) and then coverslipped with ProLong Glass Antifade Mountant (ThermoFisher). Maximum projection Z-stack and z-plane confocal images were obtained using a Leica TCS SPE laser scanning confocal microscope with a 40× objective (NA 1.15) utilizing the 405-, 488-, and 635-nm lasers to visualize DAPI, Alexa Fluor 488, and Alexa Fluor 647, respectively. Images were processed using the Leica AF software and Adobe Photoshop software.

### Drosophila husbandry

All fly lines were maintained at 25°C. The steroid mifepristone (RU486, Sigma-Aldrich, St. Louis, MO) was used to induce expression in the GeneSwitch driver system (20). RU486 was prepared at 4 mg/mL in ethanol, aliquoted, and stored at −20°C. The working RU486 concentration in the fly media was 20 μg/mL. Adult males were collected within 48 hours of eclosion and added to RU486-containing vials for 120 hours to induce polyPR expression. IU1 (Sigma-Aldrich) was reconstituted in ethanol and added to the fly media at a final concentration of 100 μM.

### Immunofluorescence of *D. melanogaster* brains

Adult male flies were collected 120 hours post RU486 exposure. Brains were dissected and fixed using 200 μL of 4% paraformaldehyde for 50 minutes, in accordance with previous literature (21). For fixation and all subsequent steps, tubes/slides were kept on a nutator to ensure samples were properly washed with solution. Following fixation, 4% paraformaldehyde was removed, and samples were washed 3 times with 200 μL of phosphate-buffered saline (PBS). For blocking, samples were incubated in 10% normal goat serum (Thermo Fisher Invitrogen) in PBS for 10 minutes and then washed 3 times with 200 μL of PBS. Following blocking, brains were incubated with primary antibody against PSMA1 [26S Proteasome α Antibody (IIG7), Santa Cruz Biotechnology, Dallas, TX, 1:100 dilution], FLAG (Monoclonal ANTI-FLAG M2 antibody, Sigma-Aldrich, 1:1000 dilution), or ubiquitin [Anti-Ubiquitin Antibody (P4D1, Santa Cruz Biotechnology, 1:500 dilution) in PBS] with 1.5% normal goat blocking serum overnight at 4°C. In the following day, samples were removed from 4°C and washed with 3 changes of PBS for 5 minutes each. Samples were then protected from light and incubated with secondary antibody for 60 minutes at room temperature [ThermoFisher Invitrogen goat anti-mouse IgG (H + L) highly cross-adsorbed secondary antibody carrying Alexa Fluor 594; diluted to 5.0 μg/mL in PBS with 1.5% normal goat serum]. Following secondary incubation, samples were washed 3 times with PBS for 5 minutes each and then immediately mounted and imaged. After mounting the samples on a glass microscope slide, a large drop of DAPI mounting medium (Santa Cruz Biotechnology UltraCruz Hard-set Mounting Medium with DAPI) was then added to the sample as appropriate. Images were collected using a Nikon Eclipse 80i confocal microscope and the EZ-C1 program. Focus and gain were adjusted to reduce background fluorescence and increase sample clarity.

### LysoTracker assay

Brains were dissected in PBS and stained for 2 minutes with LysoTracker Deep Red (Invitrogen) diluted in PBS (final concentration of 500 nM). Samples were mounted in PBS and immediately visualized by confocal microscopy.

### Lysate preparation

To prepare samples, adult male flies were collected 120 hours post-RU486 exposure (20 μg/mL). For each lane to be run in sodium dodecyl sulfate-polyacrylamide gel electrophoresis (SDS-PAGE), 4 adults were used. Their heads and wings were removed, and they were stored in microfuge tubes at −80°C. For lysis, prepped adults were removed from −80°C freezer and allowed to thaw for 30 minutes before being washed twice in 1 mL lysis buffer containing 100 mM 4-(2-hydroxyethyl)-1-piperazineethanesulfonic acid (HEPES), pH 7.3, 200 mM KCl, 2 mM ethylene glycol-bis(β-aminoethyl ether)-*N*,*N*,*N*′,*N*′-tetraacetic acid (EGTA), and 1 mM DTT. Following these initial washes, adults were suspended in 300 μL of fully supplemented lysis buffer (100 mM HEPES, pH 7.3; 200 mM KCl, 2 mM EGTA, 1 mM DTT, 100 mM phenylmethylsulfonyl fluoride [PMSF] in isopropanol, protease inhibitor cocktail [PICS]) and flash frozen in liquid nitrogen 3 times and allowed to thaw for 45 minutes on ice. For ubiquitin blotting, 50 mM of *N*-ethylmaleimide (NEM; Sigma) was added to the lysis buffer. Samples were then transferred to a glass homogenizer and homogenized with approximately 50 twists, rested on ice for 5 minutes, and then homogenized with an additional 10 twists. The sample was then moved to a new microfuge tube and allowed to gravity sediment for 60 minutes on ice. The resulting lysate supernatant was transferred to a fresh tube, labeled, and stored at −80°C for future use in experiments. Pierce Coomassie Plus (ThermoFisher) was used to determine the protein concentration of each lysate to determine the amount of sample used in co-immunoprecipitation (Co-IP) and Western blotting.

### Co-immunoprecipitation

For optimal yield in Co-IP, antibodies were mixed directly with protein sample prior to addition of beads rather than being crosslinked to beads and then added to sample. On ice, 10–50 μg of cell lysate was mixed with the recommended amount of antibody (26S Proteasome α Antibody [IIG7] sc-65755, Santa Cruz Biotechnology) and incubated rocking overnight at 4°C. Following antibody incubation, 70–100 μL of recombinant Protein G-coupled Sepharose 4B beads (ThermoFisher Invitrogen) were added to the lysate, and the sample was incubated by rocking for 4 hours at 4°C. The sample was then centrifuged at 5000 rpm for 2 minutes at 4°C. The supernatant was removed and discarded, and the bead:antibody:protein complex was washed 3 times with freshly made fully supplemented lysis buffer (100 mM HEPES, pH 7.3, 200 mM KCl, 2 mM EGTA, 1 mM DTT, 100 mM PMSF in isopropanol, PICS). For each wash, the beads were mixed gently with lysis buffer, centrifuged at 5000 rpm for 2 minutes at 4°C, and the supernatant was discarded. After the final wash, samples were eluted in sodium dodecyl sulfate (SDS) buffer. For the elution step, 50 μL of protein bound to beads was eluted by adding 50 μL of 2× SDS loading buffer without DTT to the sample. The sample was boiled for 10 minutes at 50°C and pelleted, and the supernatant was transferred to a new tube, where DTT was added at a final concentration of 1 mM. Following elution, samples were boiled at 100°C for 5 minutes and then analyzed by Western blot. For 5% SDS Western blot, 3 μL of eluted sample was supplemented with 4.5 μL 2× sample loading buffer and 2.5 μL 20% SDS and then boiled at 100°C for 5 minutes.

### Western blotting

Following sample preparation, samples were loaded onto 12% precast polyacrylamide gels (Bio-Rad, Hercules, CA) and run at 120 V for 1 h at 4°C. A polyvinylidene fluoride membrane was activated with methanol, and after electrophoresis was complete, gels were transferred to the membrane at 40 mAmps overnight at 4°C. Blots were next blocked in Blotto (5% nonfat powdered milk in PBS-Tween) for 30 minutes on a rocking platform and then washed twice with PBS-Tween. Blots were probed with the appropriate dilution of primary antibody prepared in 5 mL PBS-Tween + 1% bovine serum albumin (BSA) (Sigma Monoclonal ANTI-FLAG M2 antibody produced in mouse, F1804, 1:1000; Santa Cruz Biotechnology 26S Proteasome α Antibody [IIG7], sc-65755, 1:1000; Santa Cruz Biotechnology Anti-Ubiquitin Antibody [P4D1], sc-8017, 1:500) rocking overnight at 4°C. Following primary antibody incubation, blots were blocked in Blotto for 30 minutes with rocking at room temperature and then washed 3 times in PBS-Tween. Blots were next incubated with rocking with the appropriate dilution of horseradish peroxidase (HRP)-linked secondary antibody prepared in Blotto (Abcam Goat Anti-Mouse IgG H&L, ab205719, 1:10 000; Cell Signaling Technology anti-rabbit IgG HRP-linked, CST 7074, 1:10 000) for at least 30 minutes at room temperature. Blots were then rinsed in PBS-Tween, washed 4 times with PBS-Tween on a rocking platform, and then imaged by chemiluminescence using a FluorChem HD2 Imager (Alpha Innotec, San Leandro, CA).

### Data analysis

Statistical analysis was performed using GraphPad Prism 9. For 2-group comparison, 2-tailed Student t-tests were used as indicated; for comparing more than 2 datasets, 1-way analysis of variance (ANOVA) tests, followed by Tukey’s multiple comparisons, were used. Pearson correlation coefficients were computed to assess correlations.

### Consent

Human brain specimens were obtained in accordance with the University of Pennsylvania Institutional Review Board guidelines. Where possible, pre-consent during life and, in all cases, next-of-kin consent at death was given.

## RESULTS

### PolyPR sequesters proteasomes in C9ALS patient tissue

**Figure 1.**
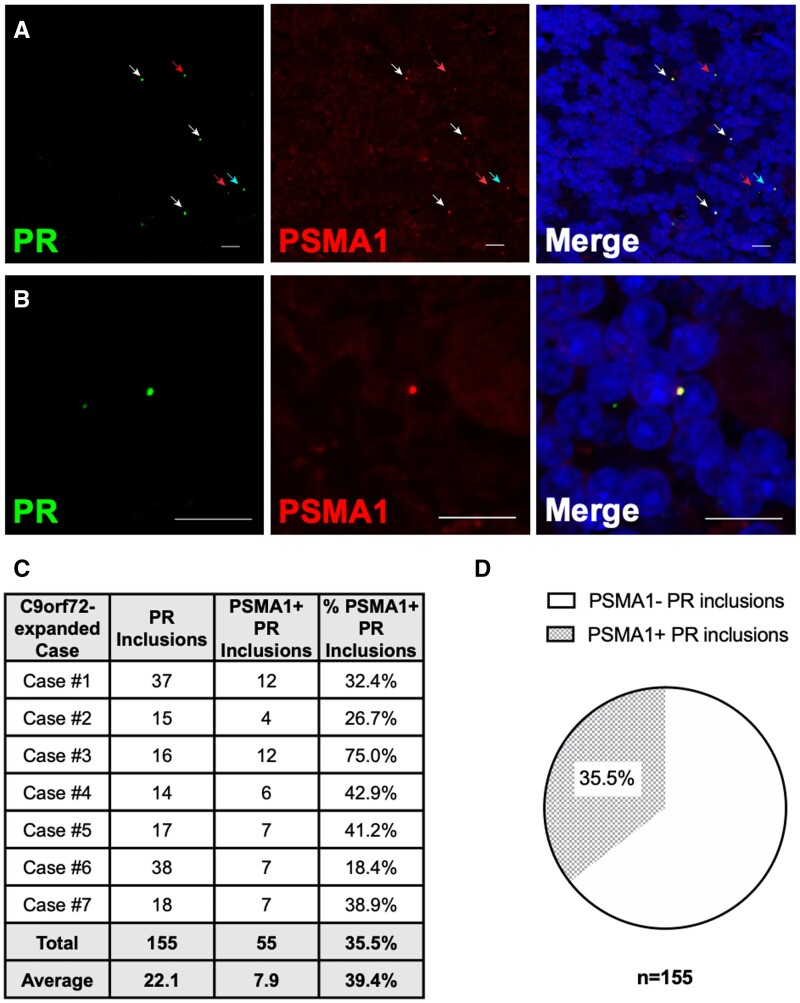
PR dipeptide sequesters the proteasome in C9ALS patient tissue. **(A)** Double immunofluorescence staining of cerebellum (granular layer) from cases with the *C9orf72*-expansion mutation detects colocalization of PR inclusions (arrows) and PSMA1. White/cyan arrow, PSMA1-positive PR inclusion; red arrow, PSMA1-negative PR inclusion. **(B)** Maximum projection Z-stack image of the inclusion in **(A)** designated with a cyan arrow showing a PR inclusion with PSMA1 colocalization. **(C** and **D)** Quantification of PR inclusions with and without PSMA1 colocalization in 7 *C9orf72* mutation cases (3 microscopic fields per case). Scale bars: 10 µm.

PolyPR DPRs have previously been shown to directly interact with and inhibit the 26S proteasome in vitro (15). However, the interaction between polyPR and the proteasome in clinical samples is yet to be reported. To confirm the presence of such interaction in patient tissue and to establish its clinical relevance, we performed dual immunofluorescence staining of cerebellum (granular layer) from 7 FTLD/ALS cases with the *C9orf72*-expansion mutation. To detect polyPR, we used a highly specific rabbit polyclonal antibody (Sigma Aldrich ABN1354), which targets a polyPR sequence; to detect 26S proteasome, we used a mouse monoclonal antibody that targets proteasome subunit alpha type-1 (PSMA1), a core component of the 26S proteasome complex and a target shown to bind arginine-rich sequences (13, 14, 17, 18). We identified positive colocalization between polyPR and proteasome in the form of inclusion-like puncta (Fig. 1A, B). This observation suggests a physical interaction between polyPR and proteasome in patients. Strikingly, we observed polyPR-proteasome interaction in every C9ALS case examined, indicating that a polyPR-proteasome interaction is a non-idiosyncratic disease feature shared across patients (Fig. 1C). Additionally, 35.5% of all polyPR inclusions examined contain proteasomes (Fig. 1C, D). The high frequency of polyPR-proteasome interaction indicates that such interaction is a prevalent cellular event in C9ALS patients. Together, our results support the interaction between polyPR and the 26S proteasome as a common, clinically relevant phenomenon in C9ALS and warrants further investigation into the effect of polyPR on proteasomal activities.

### PolyPR interacts with proteasomes in a *Drosophila* model of C9ALS

**Figure 2.**
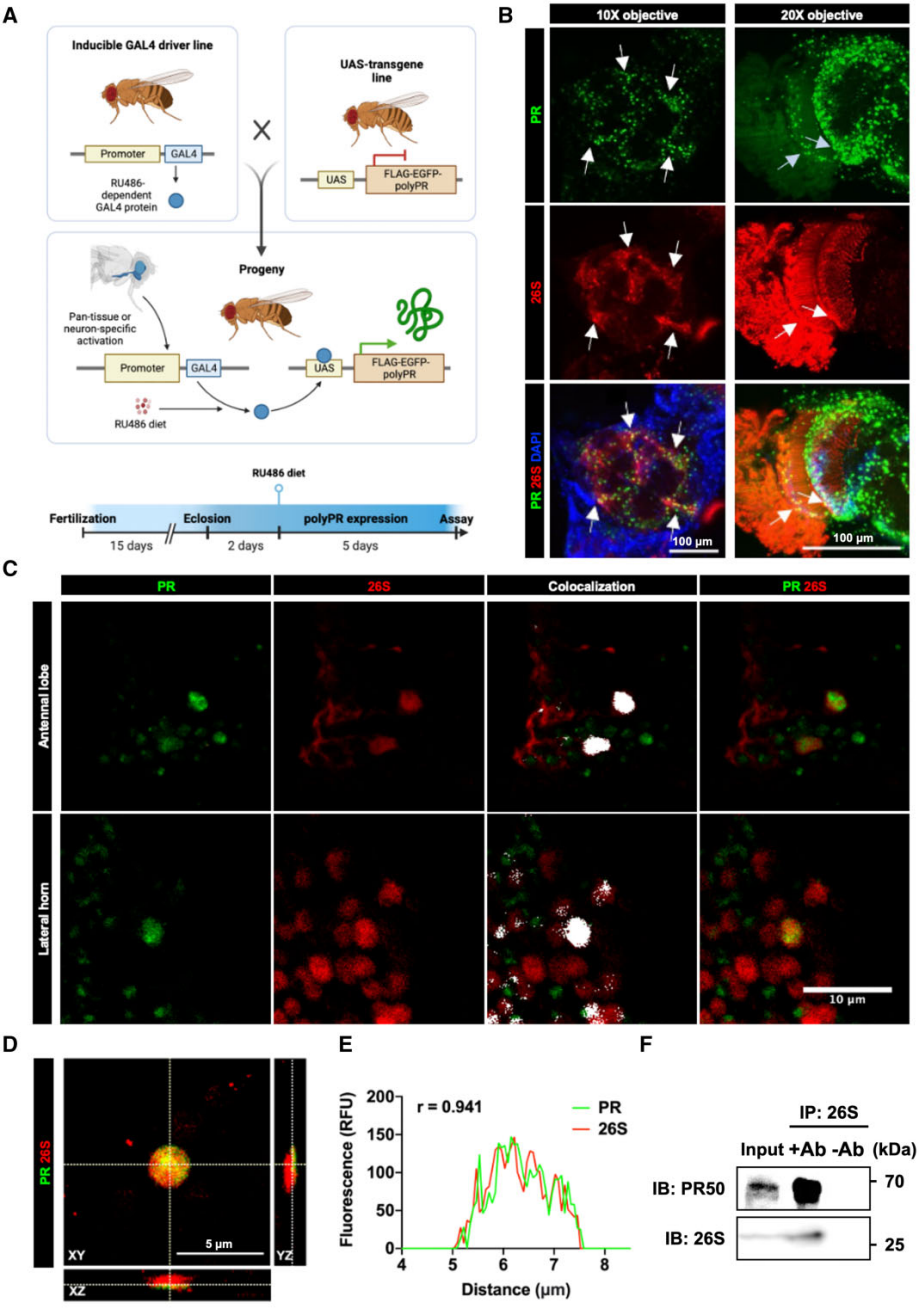
Colocalization and co-immunoprecipitation of PR50 and 26S proteasome in adult *Drosophila* brains**. (A)** Scheme for drug inducible, tissue-specific expression of PR50 in adult *Drosophila*. **(B)** Two representative maximum projection Z-stack images showing pan-tissue expression of FLAG-eGFP-tagged PR50 in adult *Drosophila* brain using the *daughterless* GeneSwitch driver. PR refers to PR50-eGFP fluorescence and 26S refers to Alexa Fluor 594-stained proteasome alpha-chain. The proteasome signal is relatively diffuse, with stronger signal present in central and optic lobes. Merged images show colocalization of polyPR and proteasomes in both central and optic lobes. Areas of colocalization are indicated with white arrows. Brains were imaged at 10× and 20×. **(C)** Pan-neuronal expression of FLAG-eGFP-tagged PR50 in adult *Drosophila* brain using the *elav* GeneSwitch driver. White, areas of fluorescence signal colocalization identified by automated image segmentation in ImageJ. **(D)** Orthogonal view in 3D space. Scale bar: 5 µm. **(E)** Fluorescence intensity profile along the marked *x*-axis at the indicated z-depth in **(D)**. Pearson correlation calculated between PR and 26S signals, *r*(198) = 0.9411, p < 0.001. **(F)** FLAG-tagged PR50-eGFP coimmunoprecipitated with 26S proteasome. Ab, antibody; IB, immunoblotting; IP, immunoprecipitation; PR, proline-arginine. −Ab: mock IP experiment assembled with only the 26S antibody absent to control for nonspecific binding to Protein G-coupled Sepharose beads. Supplementary DataFigure S2 shows uncropped versions of these blots.

We used a transgenic *Drosophila* model overexpressing polyPR to further interrogate the effects of polyPR dipeptide on proteasomal activity in vivo*.* Widely used to study FTLD/ALS, this fly model exhibits shortened lifespan, impaired locomotion and development, and severe neurodegeneration, which replicates C9ALS-associated phenotypic profiles (9, 22–25). We initially expressed a FLAG-enhanced green fluorescence protein (eGFP)-tagged 50-repeat-long PR construct (PR50) (9) using a pan-tissue *daughterless* driver with an inducible GeneSwitch promoter (20) to induce PR50 expression upon eclosion to avoid the larval and developmental toxicity of PR50 (Fig. 2A and Supplementary DataFig. S1) (9). The initial rationale for using the *daughterless* driver was to assume that colocalization of PR50 with the proteasome would likely be independent of tissue type. Fly brains were then removed and stained for proteasomes to assess colocalization of polyPR and proteasomes. To probe for the 26S proteasome in *Drosophila*, we used a mouse monoclonal antibody raised against the α-subunit of the 20S catalytic core of the *Drosophila* 26S proteasome. PolyPR expression was seen distributed throughout the brain, as imaged using a confocal microscope, indicating that the GeneSwitch driver and RU486 were successful in inducing polyPR expression (Fig. 2B). Proteasome signal was relatively diffuse and present throughout the entire brain, although higher levels of expression were observed in the central brain and the optic lobes. PolyPR and proteasomes are present in the same tissues, supporting the possibility of their interaction in vivo.

To investigate neuronal-specific effects, we also expressed the transgenes by pan-neuronal expression using the *elav* GeneSwitch driver. Immunofluorescence microscopy showed that FLAG-eGFP-tagged PR50 colocalizes with proteasomes in a subset of cells across anatomical regions, including lateral horns and antennal lobes (Fig. 2C–E). Additionally, FLAG-eGFP-tagged PR50 coimmunoprecipitated with the α-subunit of the *Drosophila* proteasome, indicating that PR50 and proteasome interact directly, suggesting an interacting complex in *Drosophila*, similar to that shown in in vitro work (Fig. 2F and Supplementary DataFig. S2) (15). Overall, our results support the physical interaction of the proteasome by PR50 in *Drosophila*.

### PolyPR causes the accumulation of ubiquitinated proteins in *Drosophila*

**Figure 3.**
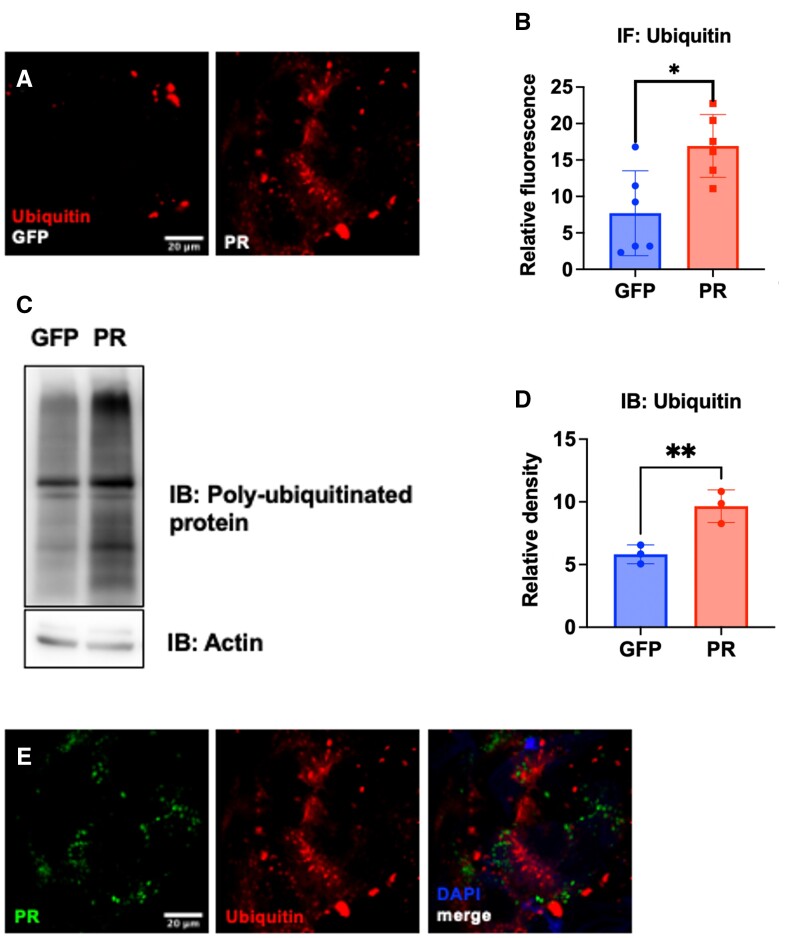
PR50 causes the accumulation of ubiquitinated proteins in *Drosophila*. **(A)** Ubiquitin accumulates in PR50-expressing brains. **(B)** Quantitative analysis of the fluorescence microscopy in **(A)**. n = 6 for each condition; *p < 0.05; **p < 0.01 by Student t-test. Whole-mount confocal images of the brains were Z-stack collapsed and analyzed for integrated fluorescence intensity in ImageJ. The integrated intensity from each brain is a single data point in the statistical test. **(C)** Blots stained for ubiquitin of PR50-expressing and control brains. PR50 expression increases poly-ubiquitinated protein level. **(D)** Quantitative analysis of the Western blots normalized against actin in **(C)**. n = 3; *p < 0.05; **p < 0.01 by Student t-test. **(E)** Ubiquitin does not colocalize with PR50, indicating that overexpressed PR50 is not the main substrate for ubiquitination and that the ubiquitination of PR50 is not the culprit for ubiquitin accumulation.

Proteasomes efficiently degrade polyubiquitinated proteins, and the inhibition of the proteasome can cause the accumulation of ubiquitin conjugate (26, 27). To test the hypothesis that polyPR inhibits proteasomal activity in *Drosophila*, we measured the level of ubiquitin and ubiquitinated proteins in fly brains expressing PR50. To account for the effect of overexpressing a foreign protein, we compared PR50-expressing flies with eGFP-expressing flies as a control. Immunofluorescence staining against ubiquitin shows an accumulation of ubiquitin in PR50-expressing flies (Fig. 3A, B). To biochemically distinguish ubiquitin monomers from conjugated ubiquitin, we separated fly head lysates via SDS-PAGE and probed for ubiquitin. The ubiquitin blot shows an elevated level of polyubiquitinated proteins in PR50-expressing flies, in agreement with proteasomal inhibition and a disruption in proteasomal flux (Fig. 3C, D). An alternative explanation for the accumulation of ubiquitinated proteins is that the overexpression of PR50 provides additional substrates for ubiquitination. However, PR50 does not colocalize with ubiquitin, indicating that overexpressed PR50 is not the main substrate for ubiquitination and that the ubiquitination of PR50 is not the culprit for the accumulation of ubiquitinated proteins (Fig. 3E). Overall, our results show that polyPR causes the accumulation of ubiquitinated proteins in *Drosophila*, in agreement with proteasomal inhibition.

### PolyPR induces lysosomal accumulation and hyper-acidification, which is reversible by a proteasomal enhancer

**Figure 4.**
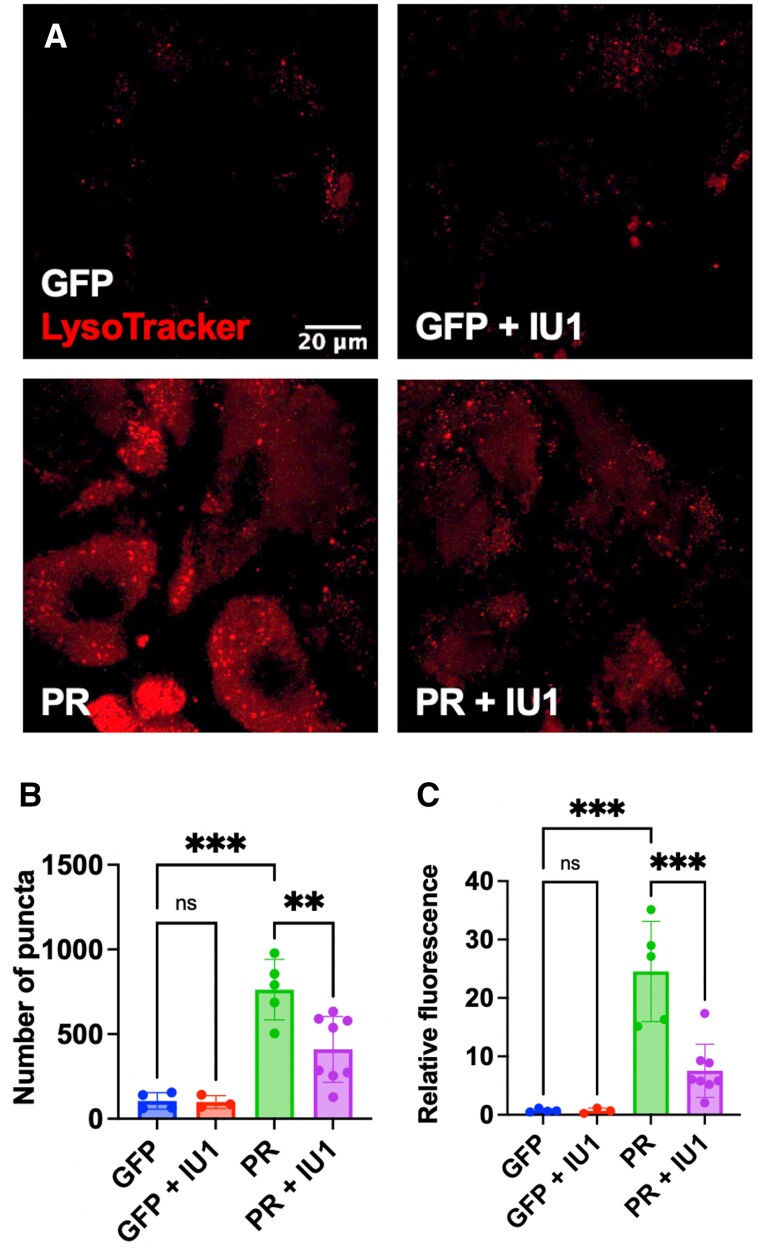
PR50 induces lysosomal accumulation and acidification, which is reversible by a proteasomal enhancer. **(A)** Acidic lysosomal organelles accumulate in PR50-expressing brains as indicated by a LysoTracker dye. The accumulation of lysosomes is rescued by a proteasomal enhancer IU1. **(B)** Quantitative analysis of lysosome numbers in **(A)**. n > 3; *p < 0.05; **p < 0.01; ***p < 0.001 by 1-way ANOVA followed by Tukey’s multiple comparisons. **(C)** Quantitative analysis of LysoTracker fluorescence in **(A)**. n > 3; *p < 0.05; **p < 0.01; ***p < 0.001 by 1-way ANOVA followed by Tukey’s multiple comparisons. ANOVA, analysis of variance.

The UPS and autophagy-lysosomal pathway are the 2 major degradative and quality control mechanisms in eukaryotes. The impairment of the UPS is often compensated by the upregulation of autophagy (28). Our work suggests that the polyPR inhibits proteasomal function in *Drosophila*, which raises the question as to whether autophagy and lysosomal degradation are upregulated to compensate for the impaired UPS. We used LysoTracker Red, a dye that selectively labels acidic organelles, to monitor lysosomal acidification as a proxy for lysosomal activity. We observed a significant increase in overall fluorescence and the number of acidified organelles in PR50-expressing brains, indicating that PR dipeptide induces lysosomal accumulation and hyper-acidification, in agreement with a compensatory lysosomal response to proteasomal impairment (Fig. 4A–C). To confirm whether the change in lysosomal activity is indeed induced by the impairment of the proteasome by polyPR, we administered a highly selective proteasomal small-molecule enhancer, IU1, to PR50-expressing flies (29). IU1 enhances proteasomal activity by inhibiting Usp14, a proteasome-associated deubiquitinating enzyme, which inhibits the degradation of ubiquitin-protein conjugates. The administration of IU1 successfully reversed the accumulation and hyper-acidification of lysosomes, suggesting the lysosomal phenotypes seen in PR50-expressing flies are contingent upon proteasomal inhibition (Fig. 4A–C). Taken together, these results indicate that PR-induced proteasomal inhibition perturbs lysosomal activity, likely as a compensatory response.

## DISCUSSION

Previous in vitro work has shown that the mutant *C9orf72* gene product, PR20 (but not GR20), interacts with the proteasome and impairs its activity. Here, we show that this interaction is found in human diseased brain tissue from C9ALS patients and influences the expression of human genes involved in both the UPS and autophagy-lysosomal pathways, demonstrating that this interaction is potentially of concern in disease etiology. Our in vivo transgenic *Drosophila* studies confirm the colocalization of the polyPR DPR with the α-subunit of the proteasome. We demonstrated through co-IP experiments that this interaction is physical. We have shown that polyPR DPR expression in *Drosophila* neurons results in an accumulation of ubiquitin, as shown in earlier in vitro work (15).

**Figure 5.**
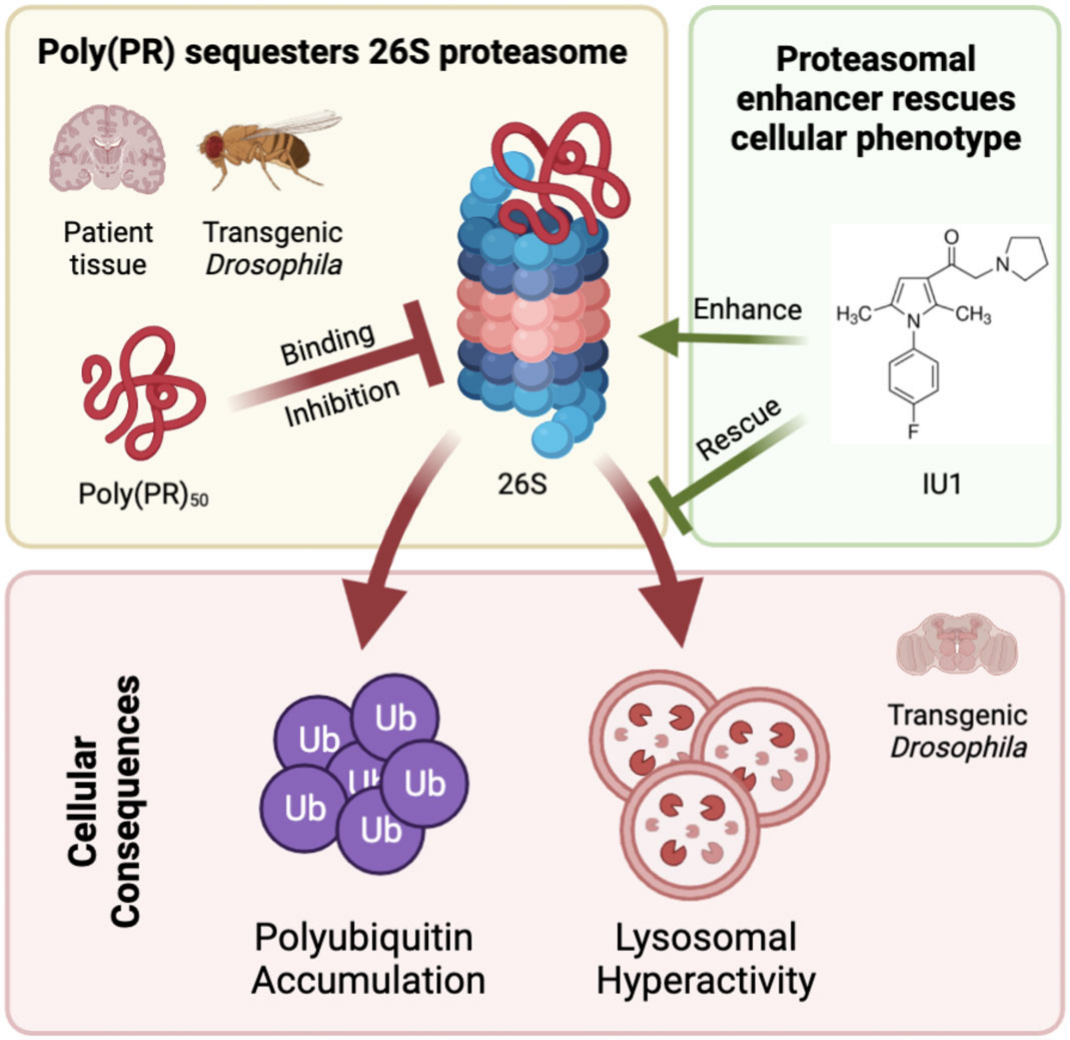
Schematic diagram showing the major findings from this study and implications for the molecular basis of disease for *C9orf72* in ALS patients. Created with BioRender.

Furthermore, we show that polyPR DPR proteasome inhibition causes lysosomes to accumulate and hyper-acidify in the *Drosophila* brain. Abnormal lysosomal pH impairs lysosomal degradation, cargo loading, catabolite export, vesicle movement, and nutrient sensing and is involved in multiple neurodegenerative diseases (30–32). Interestingly, PR-induced lysosomal abnormalities can be reversed by a proteasomal activator, IU1, suggesting that the observed lysosomal abnormalities—at least partially—occur downstream of and are regulated by proteasomal activities. This is consistent with previous findings that the autophagy-lysosomal pathway is activated to compensate for proteasomal impairment (3). Overall, we provide multiple lines of evidence to support the involvement of proteolytic dysfunctions in C9ALS pathology (Fig. 5).

While postmortem studies of patient tissue have revealed low concentrations of polyPR aggregates in comparison to other DPRs, it is important to note that these tissues represent end-stage disease and are not necessarily reflective of early disease progression (4). Postmortem studies may additionally be limited in that they rely on the detection of large inclusions through immunohistochemistry in fixed tissues (33). While the more hydrophobic DPRs—polyGA, polyGP, and polyPA—are aggregation-prone and thus easily detectable, polyPR is positively charged, making it more soluble and less likely to self-aggregate (34). This higher solubility may be important for several reasons. First, a growing body of evidence suggests that soluble proteins may be more toxic than their insoluble aggregate counterparts in neurodegenerative diseases (35). Second, soluble species are more difficult to detect through traditional immunohistochemistry (33). Finally, the high solubility and positive charge of polyPR may allow it to easily enter cell nuclei and lead to rapid toxicity and cell death, which may result in a paucity of polyPR-positive cells in postmortem tissues (34). Thus, it is imperative to investigate the exact method through which polyPR toxicity occurs and how it relates to the overall pathogenesis of ALS.

It remains to be determined to what degree proteasome disruption contributes to the toxicity observed in neuronal cells when compared to other mechanisms, such as interference with RNA granules (36), nuclear-cytoplasmic transport (33, 37), or mitochondrial dysfunction (38). It will be important to establish to what extent these different modes of interference affect the pathology of C9ALS (39), and how this reconciles with the more recent finding of haploinsufficiency in the normal function of *C9orf72* in the regulation of autophagy (40).

## CONSENT FOR PUBLICATION

All authors have reviewed the contents of the manuscript being submitted, approved of its contents, validated the accuracy of the data, and consented to publication.

## Supplementary Material

nlad078_Supplementary_Data
