# Supplementary material for: C9orf72 proline-arginine dipeptide repeats disrupt the proteasome and perturb proteolytic activities
Source: J Neuropathol Exp Neurol. 2023 Oct 3;82(11):901–10. doi: 10.1093/jnen/nlad078 (PMC10587997; doi:10.1093/jnen/nlad078)
Supplement: nlad078_Supplementary_Data [file nlad078_supplementary_data.docx]

**Supplementary Information**


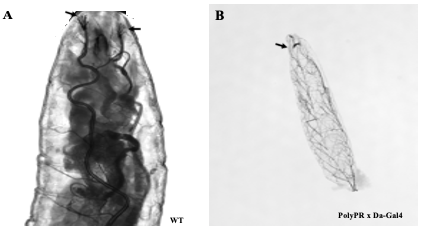


**Figure S1. Toxicity of PR50 in *Drosophila* larvae.** DIC images collected using the Nikon Eclipse 80i confocal microscope and the NIS-Elements program. (A) Wild-type W1118 larva. (B) Larva with polyPR driven by the ubiquitous Da-Gal4 driver. Arrows indicate spiracles. All larvae are third instar or the latest developmental stage reached by cross offspring. All images taken at 40x magnification and consistently scaled.


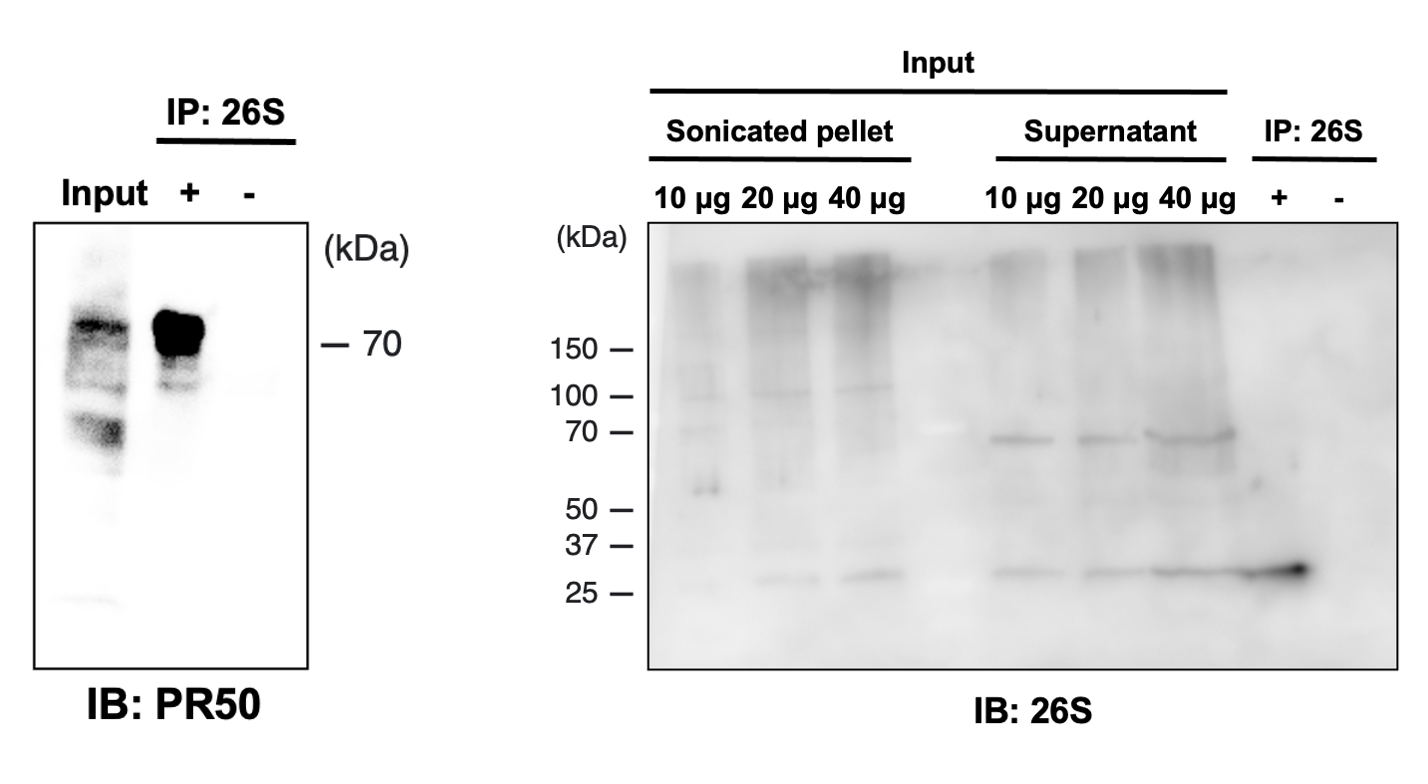


**Figure S2.** Uncropped blot of PR50-proteasome co-immunoprecipitation and Western analysis of co-immunoprecipitation fractionation steps. 26S pull down probe for PR50. 26S probe. Same samples were run for both blots.
